# miR-155-5p modulates malignant behaviors of hepatocellular carcinoma by directly targeting CTHRC1 and indirectly regulating GSK-3β-involved Wnt/β-catenin signaling

**DOI:** 10.1186/s12935-017-0469-8

**Published:** 2017-12-08

**Authors:** Gang Chen, Dongdong Wang, Xiongqi Zhao, Jun Cao, Yingpeng Zhao, Fan Wang, Jianhua Bai, Ding Luo, Li Li

**Affiliations:** Department of Hepatobiliary Surgery, First People’s Hospital of Kunming City, No. 504 Qinnian Road, Kunming, 650034 Yunnan China

**Keywords:** Hepatocellular carcinoma (HCC), Malignant behaviors, microRNA-155-5p (miR-155-5p), Collagen triple helix repeat containing 1 (CTHRC1), GSK-3β-involved Wnt/β-catenin signaling

## Abstract

**Background:**

Hepatocellular carcinoma (HCC) remains one of the most lethal cancers. MicroRNA-155 (miR-155) and collagen triple helix repeat containing 1 (CTHRC1) were found to be involved in hepatocarcinogenesis, but their detailed functions in HCC are unclear. Here, we aimed to investigate the underlying role of miR-155-5p and CTHRC1 in HCC.

**Methods:**

miR-155-5p and CTHRC1 expression levels were detected by qRT-PCR, IHC and WB in HCC patients and cell lines. Dual-luciferase assay, qRT-PCR and WB were used to validate the target interaction between miR-155-5p and CTHRC1. Biological behaviors, including apoptosis, cell cycle progression, and cell proliferation, invasion and migration, were measured by flow cytometry, CCK-8 assay and Transwell tests. A xenograft model was established to examine the effects of miR-155-5p and CTHRC1 on tumor formation. WB was finally utilized to identify the role of GSK-3β-involved Wnt/β-catenin signaling in HCC growth and metastasis.

**Results:**

Our results showed that miR-155-5p and CTHRC1 were down-regulated and up-regulated, respectively, in HCC patients and cell lines. Dual-luciferase assay verified that CTHRC1 was the direct target of miR-155-5p. Moreover, elevated miR-155-5p expression promoted apoptosis but suppressed cell cycle progression and cell proliferation, invasion and migration in vitro and facilitated tumor formation in vivo; elevated CTHRC1 expression abolished these biological effects. Additionally, miR-155-5p overexpression increased metastasis- and anti-apoptosis-related protein expression and decreased pro-apoptosis-related protein expression, while forced CTHRC1 expression conserved the expression of these proteins.

**Conclusion:**

Altogether, our data suggested that miR-155-5p modulated the malignant behaviors of HCC by targeting CTHRC1 and regulating GSK-3β-involved Wnt/β-catenin signaling; thereby, miR-155-5p and CTHRC1 might be promising therapeutic targets for HCC patients.

**Electronic supplementary material:**

The online version of this article (10.1186/s12935-017-0469-8) contains supplementary material, which is available to authorized users.

## Introduction

Liver cancer has been one of the most fatal and prevalent malignant tumors in the human population worldwide, and hepatocellular carcinoma (HCC) is the most common type of liver cancer, accounting for over 90% of all cases [1]. An epidemiological survey revealed that the most prominent risk factors for HCC development include chronic hepatitis B and C viral infection, chronic alcohol consumption, aflatoxin-B1-contaminated food uptake and metabolic disorders, such as hemochromatosis and α1-antitrypsin deficiency [2, 3]. Moreover, recent studies reported that there are approximately 1,000,000 new cases of HCC per year, with the incidence equal to the mortality rate; thereby, HCC and its associated high rate of morbidity and mortality are still a major health problem [1]. HCC is normally asymptomatic in the early stage, where standard treatments in conventional clinical practice, such as surgical resection, local ablation, liver transplantation or targeted therapy, could cure a proportion of HCC patients and prolong their lifetime [4]. However, most patients with HCC that are exhibiting evident clinical symptoms are diagnosed at the intermediate or advanced disease stages, where satisfactory curative approaches are often not feasible [5, 6]. Therefore, early diagnosis of HCC is crucial for improving the survival rate and ameliorating the quality of life of patients.

Although the elemental understanding of the molecular mechanisms involved in hepatocarcinogenesis has rapidly increased over recent decades, effective early diagnostic technologies and systemic therapeutic strategies for this disease have not been attained [3, 7]. Thus, currently, the myriad of research has mainly focused on the identification of new, highly sensitive and specific biomarkers for the stages of initiation, promotion and progression of HCC [8]. These biomarkers might not only provide useful clinical information to help in patient management and allow more accurate predictions of the prognoses for patients with HCC but also be considered novel molecular targets for potential therapeutic agents.

MicroRNAs (miRNAs), a class of small endogenous non-coding functional RNAs of approximately 18–22 nucleotides in length, are widespread in most eukaryotic organisms [9]. These RNAs are able to act as post-transcriptional regulators to control the expression of their messenger RNA (mRNA) targets via mRNA degradation and/or translational repression in a sequence-specific manner [10]. Moreover, the interplays between miRNA and mRNA form a complex network that regulates numerous cellular processes, including the regulation of innate immune and adaptive immune responses and the modulation of tissue differentiation, cellular apoptosis, cell proliferation, signal transduction and organ development [11]. Thus, aberrant miRNA expression has been implicated in the occurrence and development of many human diseases, such as autoimmune diseases, cancer, neuro-developmental disorders and cardiovascular syndromes [12]. Accumulating evidence has demonstrated that numerous miRNAs present ectopic expression in HCC patient serum and plasma as well as in HCC cells and tissues; thereby, miRNAs may be useful as novel clinical markers for the early diagnosis, therapeutic monitoring and prognostic prediction of HCC [13]. For example, miR-122, which is highly abundant in liver, was significantly down-regulated in a large number of HCC patients, ultimately suppressing tumor invasion, proliferation and metastasis in HCC by directly binding to the 3′-UTR of the Distal-less 4 (DLX4) gene [14]; miR-21, which is markedly up-regulated in HCC, could promote tumor invasion and metastasis and accelerate tumor growth by targeting phosphatase and tensin homolog (PTEN) and programmed cell death 4 (PDCD4) [15].

Collagen triple helix repeat containing 1 (CTHRC1), initially found in a screen for differentially expressed genes in balloon-injured versus normal rat arteries, was widely detected in human solid cancers, especially cancers of the gastrointestinal tract, lung, breast, thyroid, ovarian, cervix, liver, and pancreas [16]. Nevertheless, in previous studies, it was found that CTHRC1 was overexpressed in HCC [17]; however, the detailed function of CTHRC1 in HCC remains to be elucidated. Moreover, we predicted by bioinformatics analysis that miR-155 might be an upstream regulator of CTHRC1. miR-155, produced from the processing of the B cell integration cluster (BIC), exerts an important role in various physiological and pathological processes, especially in tumorigenesis [18]. Hence, in the current study, we aimed to further investigate the role of miR-155 and CTHRC1 in the pathogenesis and progression of HCC and to clarify whether miR-155 and CTHRC1 have the potential to be new biomarkers for aggressive HCC and to be new therapeutic targets in treating HCC.

## Materials and methods

### Ethics statement and specimen collection

This study was performed under the approval of the Clinical Management Committee of First People’s Hospital of Kunming City and with written informed consent obtained from each patient before operation. HCC tissues and matched adjacent, non-tumorous normal tissues 5 cm away from the cancer lesion were collected from five HCC patients who did not receive any preoperative adjuvant therapies, such as radiotherapy, chemotherapy, and radiofrequency ablation. After the tissue specimens were removed, they were washed with a sufficient amount of cold phosphate-buffered saline (PBS) to reduce blood contamination and then immediately placed in liquid nitrogen. After operation, these specimens were transferred and stored at − 80 °C for further examination by quantitative reverse transcription-polymerase chain reaction (qRT-PCR) assay, immunohistochemical (IHC) analysis and western blotting (WB) experiments for the detection of miR-155-5p and CTHRC1.

### Cell culture

Five strains of HCC cell lines, including HCCLM3, SMMC-7721, BEL-7402, HepG2 and HuH-7, purchased from American Type Culture Collection (ATCC), were cultivated in complete culture medium containing RPMI-1640 (Gibco, USA) supplemented with 10% fetal bovine serum (FBS, Gibco, USA) plus 2 mmol/l l-glutamine, 100 U/ml penicillin and 100 µg/ml streptomycin and maintained in a humidified incubator at 37 °C with 5% CO_2_. After the cells grew along the culture flask wall, the culture medium was renewed every 1–2 days, and when cells were 90% confluent, 0.25% trypsin (Sigma, USA) was used for digestion and subculture. The expression levels of miR-155-5p and CTHRC1 were analyzed by qRT-PCR and WB assays in these HCC cell lines.

293T cells obtained from ATCC were cultured in RPMI-1640 with 10% FBS, l-glutamine and 1% penicillin/streptomycin in 37 °C incubators for dual-luciferase assay.

### qRT-PCR assay for miRNA and gene expression

For miR-155-5p analysis, the miRNA enrichment procedure was first performed using a mirVana miRNA Isolation Kit (Ambion, USA) following the manufacturer’s protocols. RNA samples were quantified using a BioPhotometer, and the integrity of the RNA was verified by agarose-formaldehyde gel electrophoresis. One microgram of RNA from each sample was reverse-transcribed with a miRNA-specific stem-loop primer using an M-MLV Reverse Transcriptase Kit (Promega, USA) in accordance with the manufacturer’s instructions. Subsequently, each complementary DNA (cDNA) was amplified on the ABI PRISM^®^ 7500 Sequence Detection System (Applied Biosystems, USA) with the corresponding miRNA primers, and the amplifications were achieved using a SYBR Green qPCR SuperMix Kit (Invitrogen, USA). The reactions were incubated in a 96-well optical plate at 95 °C for 2 min, followed by 40 cycles of 15 s at 95 °C and 32 s at 60 °C and dissociation at 95 °C for 60 s, 55 °C for 30 s and 95 °C for 30 s. U6 snRNA served as a normalization control, and the 2^−△△Ct^ method was used to evaluate relative expression. The experiment was performed in triplicate for each sample. The primers for miR-155-5p and U6 used in the qRT-PCR experiments are listed in Table 1.

**Table 1:** Sequences of primers used for qRT-PCR assays

miRNA	Primer sequences
miR-155-5p	RT primer: 5′-CTCAACTGGTGTCGTGGAGTCGGCAATTC AGTTGAGGCTGAGA-3′ Forward primer: 5′-ACACTCCAGCTGTAAACA TCCTACACTCT-3′ Reverse primer: 5′-CTCAACTGGTGTCGTGGA-3′
U6	RT primer: 5′-CTCAACTGGTGTCGTGGAGTCGGCAATTCAGTTGAGAAAAATATGG-3′ Forward primer: 5′-CTCGCTTCGGCAGCACA-3′ Reverse primer: 5′-AACGCTTCACGAATTTGCGT-3′
CTHRC1	Forward primer: 5′-ATAATGGAATGTGCTTACAAGG-3′ Reverse primer: 5′-TTCCCAAGATCTATGCCATAAT-3′
18S rRNA	Forward primer: 5′-CCTGGATACCGCAGCTAGGA-3′ Reverse primer: 5′-GCGGCGCAATACGAATGCCCC-3′

For CTHRC1 analysis, total RNA was extracted using TRIzol reagent (TIANGEN, China) as recommended by the manufacturer. Non-denaturing agarose gel electrophoresis and a BioPhotometer were used to assess the quality and quantity, respectively, of the isolated RNA. First, we reversed-transcribed individual total RNA into cDNA using an M-MLV Reverse Transcriptase Kit (Promega, USA) according to the manufacturer’s guidelines. Subsequently, the PCR reaction was conducted with the ABI PRISM^®^ 7500 Sequence Detection System (Applied Biosystems, USA) using a SYBR Green qPCR SuperMix Kit (Invitrogen, USA). The thermocycling parameters were carried out under the following conditions: predenaturation at 95 °C for 2 min, followed by 40 cycles at 95 °C for 15 s and 60 °C for 32 s, with data collection after each cycle, followed by a melting curve. The qRT-PCR primers used for CTHRC1 and 18S rRNA in this study are shown in Table 1. Samples (n = 3) were run simultaneously for each gene in triplicate, including a no-template control, and 18S rRNA was used as an endogenous control for data normalization. The relative amount of expressed mRNA was also calculated by the 2^−ΔΔCt^ method. This result is shown in Table 1.

### IHC analysis

Immunohistochemistry studies on CTHRC1 were performed on formalin-fixed, paraffin-embedded para-carcinoma tissue and carcinoma tissue sections obtained from the aforementioned patients with HCC according to the standard procedures. Briefly, the 5-μm-thick paraffin sections were deparaffinized in xylene and dehydrated with graded ethanol. After the sections were washed twice with PBS, endogenous peroxidase activity was eliminated with 3% hydrogen peroxide (H_2_O_2_) in methanol for 15 min at room temperature. Next, the slides were heated at 120 °C in a microwave oven for 20 min with citric acid buffer for antigen retrieval and then cooled for 20 min at room temperature. The slides were blocked using 2% bull serum albumin (BSA) at 37 °C for 1 h and then incubated with the primary antibodies in blocking solution against rabbit anti-CTHRC1 (1:300 dilution; Abcam, USA) overnight at 4 °C. The slides were washed with PBS three times and incubated with biotinylated goat anti-rabbit IgG at 37 °C for 30 min the following day. Subsequently, the slides were washed with PBS again and incubated with horseradish peroxidase-conjugated streptavidin for 5 min at room temperature, followed by immunodetection with diaminobenzidine (DAB). Finally, the slides were rinsed with PBS for 5 min, counterstained with Mayer’s hematoxylin for 1 min, dehydrated in a graded series of alcohol and sealed with cover slips. Images were visualized and captured under a light microscope.

### Luciferase reporter construction and luciferase assays

A fragment of CTHRC1 3′-UTR that contained the putative miR-155-5p-binding sites or a fragment of the CTHRC1 3′-UTR mutant was cloned downstream of the firefly luciferase reporter genes in the psiCHECK-2 plasmid (Promega, USA) to generate the recombinant vectors WT-CTHRC1 and Mutant-CTHRC1. Co-transfection with the recombinant vectors along with the control psiCHECK-2 plasmid (that is, the blank group), miR-155-5p mimics, miR-155-5p inhibitor, negative control (NC) plasmid or NC inhibitor were performed in 293T cells using Lipofectamine 2000. Luciferase and *Renilla* signals were measured 48 h after co-transfection using a Dual-Luciferase Reporter Assay Kit (Promega, USA) according to the manufacturer’s instructions and normalized against the activity of the *Renilla*/firefly luciferase gene. All transfection assays were tested in three independent biological replicates. The psiCHECK-2 vector, NC plasmid, NC inhibitor, miR-155-5p mimics and miR-155-5p inhibitor were purchased from Sangon Biotech, Shanghai, China.

### WB assays

WB assays were utilized to measure protein expression in this study, and all antibodies were purchased from Abcam. First, the para-carcinoma tissues and carcinoma tissues of five patients were utilized to examine CTHRC1 protein expression. Then, 293T cells transfected with the control psiCHECK-2 plasmid, NC plasmid and miR-155-5p mimics, as well as five strains of HCC cell lines, were examined for CTHRC1 protein expression. Additionally, HCCLM3 cells transfected with the NC plasmid, miR-155-5p mimics, CTHRC1-overexpression plasmid and si-CTHRC1 plasmid were used to examine β-catenin, p-GSK-3β, GSK-3β, Caspase3, Cleaved caspase3, Caspase9, Cleaved PRAP, Bax and Bak protein expression. The CTHRC1-overexpression plasmid and si-CTHRC1 plasmid were purchased from Sangon Biotech, Shanghai, China.

Three independent samples from each group were harvested and homogenized, and the cells were lysed with radioimmunoprecipitation assay (RIPA) lysis buffer containing 50 mM Tris–HCl (pH 7.5), 150 mM NaCl, 0.1% Nonidet P-40, and a mixture of protease inhibitors for 20 min on ice. After centrifugation at 12,000 rpm for 10 min at 4 °C, the insoluble material was removed, and the total protein concentration of the supernatant was measured with a BCA protein assay kit (Beyotime, China). Equal amounts of protein (30 μg) were fractionated based on their molecular weight by 8–10% sodium dodecyl sulfate-polyacrylamide gel electrophoresis (SDS-PAGE) and transferred onto polyvinylidene difluoride (PVDF) membranes at a constant current of 200 mA for 60 min. Subsequently, the membranes were blocked with 5% non-fat milk in Tris-buffered saline with 0.1% Tween-20 (TBST) for 2 h at room temperature and incubated with primary antibodies against mouse anti-GAPDH (1:10,000), rabbit anti-CTHRC1 (1:1000), rabbit anti-β-catenin (1:5000), rabbit anti-p-GSK-3β (1:500), rabbit anti-GSK-3β (1:5000), rabbit anti-Caspase3 (1:500), rabbit anti-cleaved caspase3 (1:1500), Caspase9 (1:2000), rabbit anti-cleaved PARP (1:1000), rabbit anti-Bax (1:1000) and rabbit anti-Bak (1:1000) overnight at 4 °C. After the membranes were extensively washed with TBST, they were probed with the corresponding secondary antibodies (including goat anti-mouse IgG and goat anti-rabbit IgG, 1:12,000) for 1 h at 37 °C and visualized by enhanced chemiluminescence reagents (Beyotime, China). The relative protein expression levels were analyzed using Image J software.

### Flow cytometry

Approximately 5 × 10^5^ HCCLM3 cells per well were plated in 6-well plates and cells grown to 80% confluence; then, the cells were transfected with the NC plasmid, miR-155-5p mimics, CTHRC1-overexpression plasmid and si-CTHRC1 plasmid. After 48 h, the cells with different treatments were harvested for cell apoptosis and cell cycle measurement by flow cytometry. For cell apoptosis analysis, the collected cells were washed with cold PBS, and 250 μl Annexin V binding buffer was added followed by thorough mixing. Then, the cells were stained with 5 μl Annexin V-FITC and 5 μl propidium iodide (PI) per sample for 15 min at 25 °C in the dark. Ultimately, samples were analyzed within 1 h on a FACScan flow cytometer. Cells with a combination of Annexin V-positive/PI-negative were scored as early apoptotic cells. Double-stained cells were considered to be necrotic or late apoptotic cells. Cells only stained with PI were recorded as dead cells. For cell cycle analysis, the collected cells were similarly washed with cold PBS and fixed with 1 ml ice-cold 75% ethanol at − 20 °C overnight. After centrifugation at 3000 rpm for 5 min, the ethanol was decanted thoroughly, and the cells were resuspended in 0.5 ml PI for 30 min without light. Eventually, the samples were examined with a FACScan flow cytometer, and DNA histograms were analyzed with FlowJo 7.6 software.

### Cell proliferation

Cell proliferation was determined using Cell Counting Kit-8 (CCK8) (Dojindo, Japan). In briefly, 2 × 10^4^ cells/well were seeded in 96-well plates and transfected with different plasmids, including the NC plasmid, miR-155-5p mimics, CTHRC1-overexpression plasmid and si-CTHRC1 plasmid, as described above. At the indicated time points (days 0, 1, 2, and 3), 10 μl CCK-8 reagent was added to each well, and the cells were cultured for an additional 2 h, followed by measurement of the optical density (OD) at an absorbance-wavelength of 450 nm on an enzyme immunoassay analyzer (Bio-Rad, USA). The percentage of inhibition was calculated from the following equation: (control-test/control) × 100%.

### Cell migration and invasion

Cell migration was assayed using Transwell inserts (8-μm pore filter, 24-well cell clusters; Millipore, USA) in accordance with the manufacturer’s instructions. After 48 h of transfection with the NC plasmid, miR-155-5p mimics, CTHRC1-overexpression plasmid and si-CTHRC1 plasmid, HCCLM3 cells were trypsinized, loaded into the upper chambers at a density of 2 × 10^5^ cells per well and cultured in RPMI 1640 medium supplemented with 2% FBS, while the lower chambers were filled with 500 μl complete medium containing 10% FBS as a chemo-attractant. The HCCLM3 cells were allowed to migrate from the upper to the lower chambers for 24 h at 37 °C in a humidified incubator with 5% CO_2_. Non-migratory cells above the upper chambers were removed by a cotton swab, and the cells that attached to the lower surface of the upper chambers were fixed in 4% paraformaldehyde at room temperature for 30 min and counterstained with 0.1% crystal violet for 10 min. Cells were counted from five random, non-overlapping fields for each well using ImageJ. Cell invasion assays were performed using a protocol similar to that used for the cell migration assay described above; however, Matrigel (BD Biosciences) was precoated on the upper side of the Transwell inserts, and 1 × 10^5^ cells were added per chamber.

### Nude mouse HCCLM3 cell xenograft model

BALB/c nude mice, 4–5 weeks old, were supplied by the First People’s Hospital of Kunming City. All experimental procedures were compliant with the First People’s Hospital of Kunming City and in accordance with the National Institutes of Health Guide for the Care and Use of Laboratory Animals. All mice were housed under specific pathogen-free conditions with an alternating 12 h light/dark cycle at 25 ± 2 °C and provided with free access to autoclaved food and water. After acclimatization for at least 1 week, mice were randomly divided into three groups (three mice per group): the NC group, the miR-155-5p mimic group and the CTHRC1-overexpression group. Then, each mouse was inoculated with 1 × 10^6^ transfected HCCLM3 cells by subcutaneous injection into the dorsal flanks of nude mice. The tumor size was measured every 2 days from the third day after injection by the use of a digital caliper, and the tumor volume was determined with the following formula: ${\text{tumor volume }}\left[ {{\text{mm}}^{ 3} } \right] = \left( {{\text{length }}\left[ {\text{mm}} \right]} \right) \times \left( {{\text{width }}\left[ {\text{mm}} \right]} \right)^{ 2} \times 0. 5 2$. Thirty-five days after tumor cell implantation, all mice were sacrificed by CO_2_ inhalation to ameliorate animal suffering, and tumor xenografts were excised and weighed.

### Statistical analysis

Statistical analysis was carried out using SPSS 18.0 software (IBM SPSS, USA). All data are presented as the mean ± standard deviation (SD) from triplicate experiments. Statistical significance (*p* value < 0.05) was determined among groups using one-way analysis of variance (ANOVA) followed by a Bonferroni post hoc test for multiple groups or an unpaired, two-tailed Student’s t test for two groups.

## Results

### miR-155-5p and CTHRC1 were down-regulated and up-regulated, respectively, in the carcinoma tissue of HCC patients

**Fig. 1:**
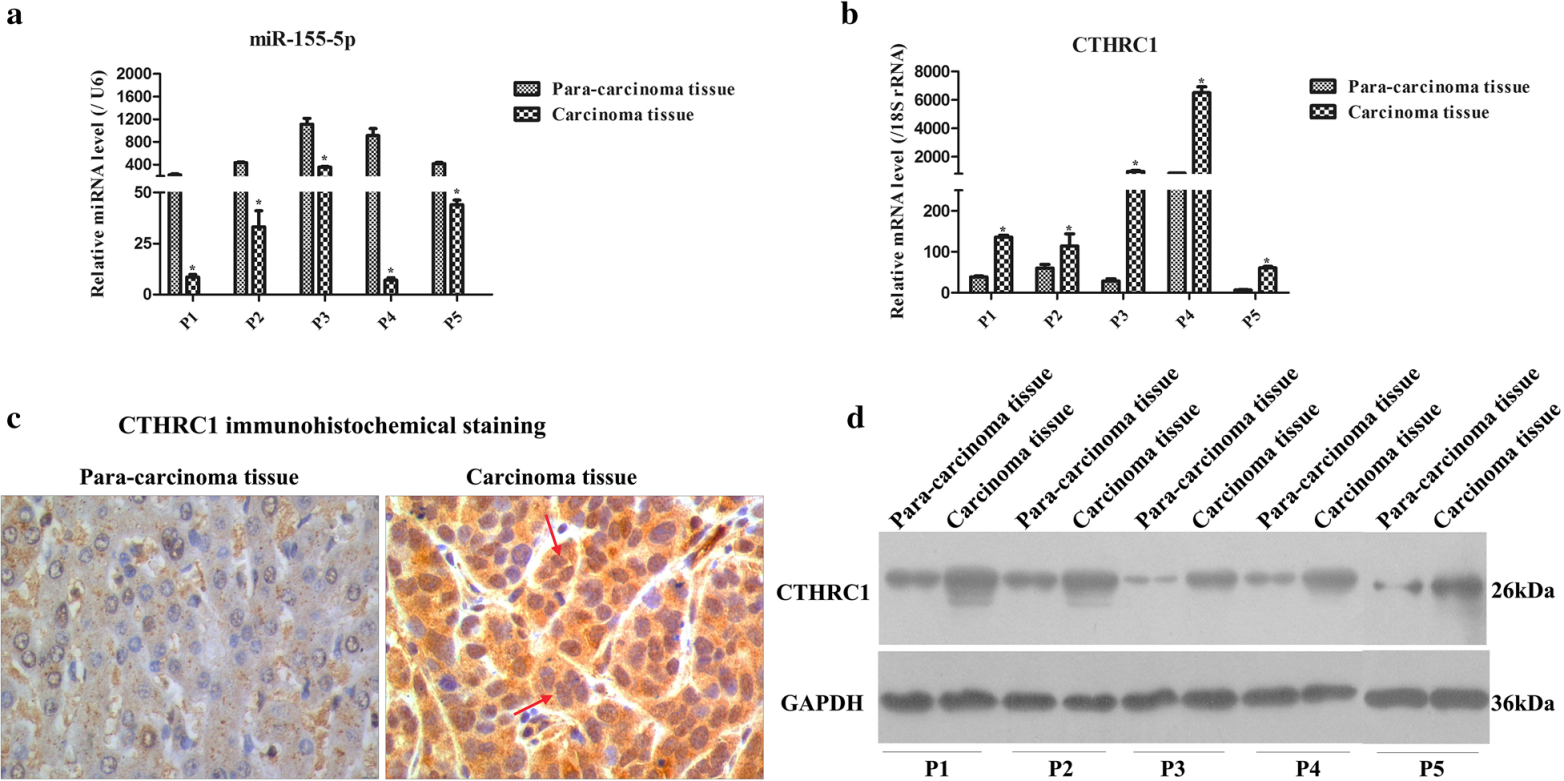
Expression of miR-155-5p and CTHRC1 in the para-carcinoma tissue and carcinoma tissue of HCC patients. **a** miR-155-5p expression was analyzed by qRT-PCR in the para-carcinoma tissue and carcinoma tissue of HCC patients. miR-155-5p was significantly down-regulated in carcinoma tissue. “p” indicates patient. *p < 0.05. **b** CTHRC1 mRNA expression was detected by qRT-PCR in the para-carcinoma tissue and carcinoma tissue of HCC patients. CTHRC1 was remarkably up-regulated in carcinoma tissue. *p < 0.05. **c** Immunohistochemical staining of CTHRC1 in the para-carcinoma tissue and carcinoma tissue of HCC patients (× 200 magnification). The red arrow represents positive staining of CTHRC1, which was mainly located in cytoplasm. **d** CTHRC1 protein expression was tested by WB in the para-carcinoma tissue and carcinoma tissue of HCC patients. CTHRC1 was notably elevated in carcinoma tissue. *p < 0.05

To investigate the expression of miR-155-5p and CTHRC1 in para-carcinoma tissue and carcinoma tissue of HCC patients, qRT-PCR was applied to detect their expression levels. As shown in Fig. 1a, b, miR-155-5p was expressed significantly higher in the para-carcinoma tissue than in the carcinoma tissue (p = 0.000 for P1, p = 0.001 for P2, p = 0.000 for P3, p = 0.000 for P4 and p = 0.000 for P5, respectively), while CTHRC1 was obviously expressed lower in the para-carcinoma tissue than in the carcinoma tissue (p = 0.001 for P1, p = 0.002 for P2, p = 0.000 for P3, p = 0.000 for P4 and p = 0.000 for P5, respectively). Moreover, IHC staining revealed that the cytoplasm of most cells in the carcinoma tissue, but not in the para-carcinoma tissue, were positively stained with CTHRC1 (Fig. 1c). Additionally, the protein level of CTHRC1 was lower in the para-carcinoma tissues than in the carcinoma tissues (Fig. 1d). Thereby, these results indicated that miR-155-5p might negatively regulate CTHRC1.

### CTHRC1 might be the direct target gene of miR-155-5p

**Fig. 2:**
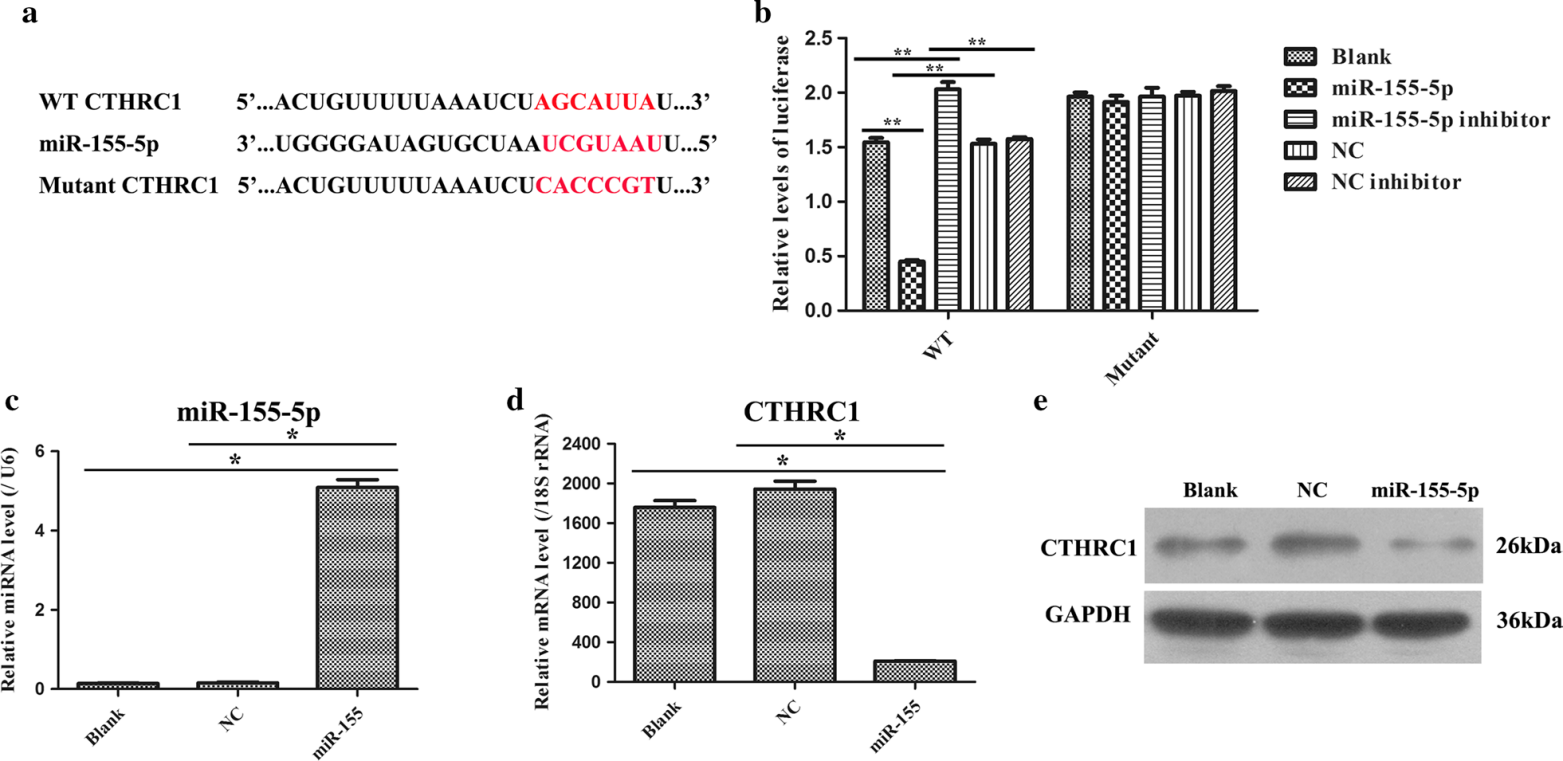
CTHRC1 is a direct target of miR-155-5p. **a** A graphical representation of miR-155-5p -binding sites in the wild-type CTHRC1-3′-UTR and mutant CTHRC1-3′-UTR. **b** Dual-luciferase reporter assay was performed in 293T cells, and the data are presented as the mean ± SD. **p < 0.01. **c** miR-155-5p expression was examined by qRT-PCR in 293T cells after transfection with NC and miR-155-5p mimic plasmids. *p < 0.05. **d** CTHRC1 mRNA expression was determined by qRT-PCR in 293T cells after transfecting with NC and miR-155-5p mimic plasmids. *p < 0.05. **e** Western blot analysis of CTHRC1 protein levels in the blank, NC and miR-155-5p mimic groups

Based on the above results, which presented an opposite expression pattern of miR-155-5p and CTHRC1 in HCC patients, we conducted a bioinformatics analysis by using TargetScan. It was predicted that miR-155-5p might be involved in regulating the gene expression of CTHRC1 (Additional file 1: Figure S1). Thereby, in this study, we further investigated this prediction by detection with the dual-luciferase reporter system. The putative miR-155-5p-binding sites of CTHRC1 and CTHRC1 corresponding mutant sites are presented in Fig. 2a. Furthermore, the data from the dual-luciferase reporter system indicated that luciferase activity was notably decreased in cells transfected with the WT-CTHRC1 vector and with the miR-155-5p mimics compared to the other four groups (p = 0.000, 0.001, 0.001 and 0.002, respectively), while the luciferase activity was not markedly changed in the cells transfected with the Mutant-CTHRC1 vector and with the five plasmids (Fig. 2b). We further examined the influence of miR-155-5p on the expression of CTHRC1 by detecting CTHRC1 mRNA and protein levels after transfection with the control psiCHECK-2 plasmid, NC plasmid or miR-155-5p mimics. After 48 h of transfection, miR-155-5p expression was measured to confirm the transfection efficiency, and it was found that the miR-155-5p mimic transfection indeed increased miR-155-5p expression (p = 0.02, 0.02, respectively) (Fig. 2c). Furthermore, the forced expression of miR-155-5p resulted in a sharp reduction in CTHRC1 mRNA and protein levels in 293T cells (p = 0.03, 0.03, respectively) (Fig. 2d, e). Hence, these data demonstrated that miR-155-5p directly targeted CTHRC1.

### The expression levels of miR-155-5p and CTHRC1 in five strains of HCC cell lines

**Fig. 3:**
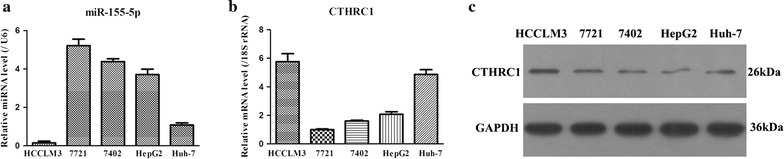
Expressions of miR-155-5p and CTHRC1 in five strains of HCC cell lines. **a** The different expression levels of miR-155-5p were analyzed by qRT-PCR in five strains of HCC cell lines. **b** The different gene expressions of CTHRC1 were measured by qRT-PCR in five strains of HCC cell lines. **c** The different protein expressions of CTHRC1 were tested by qRT-PCR in five strains of HCC cell lines

To further explore the importance of miR-155-5p and CTHRC1 in HCC, we chose to examine their expression levels in five strains of HCC cell lines. As illustrated in Fig. 3, it was observed that miR-155-5p presented the lowest expression level in HCCLM3 cells compared to the other four HCC cell lines. Moreover, both gene and protein expression levels of CTHRC1 in HCCLM3 cells were relatively higher than those in SMMC-7721, BEL-7402, HepG2 and HuH-7 cells. These data from the HCCLM3 cells were coincidently consistent with data from HCC patients. Thus, this finding implied that miR-155-5p and CTHRC1 might play a crucial role in the occurrence and development of HCC; therefore, HCCLM3 cells were selected for the following study due to their high expression levels of CTHRC1.

### miR-155-5p promoted HCCLM3 cell apoptosis and induced cell cycle arrest in G1/G0 phase probably through CTHRC1

**Fig. 4:**
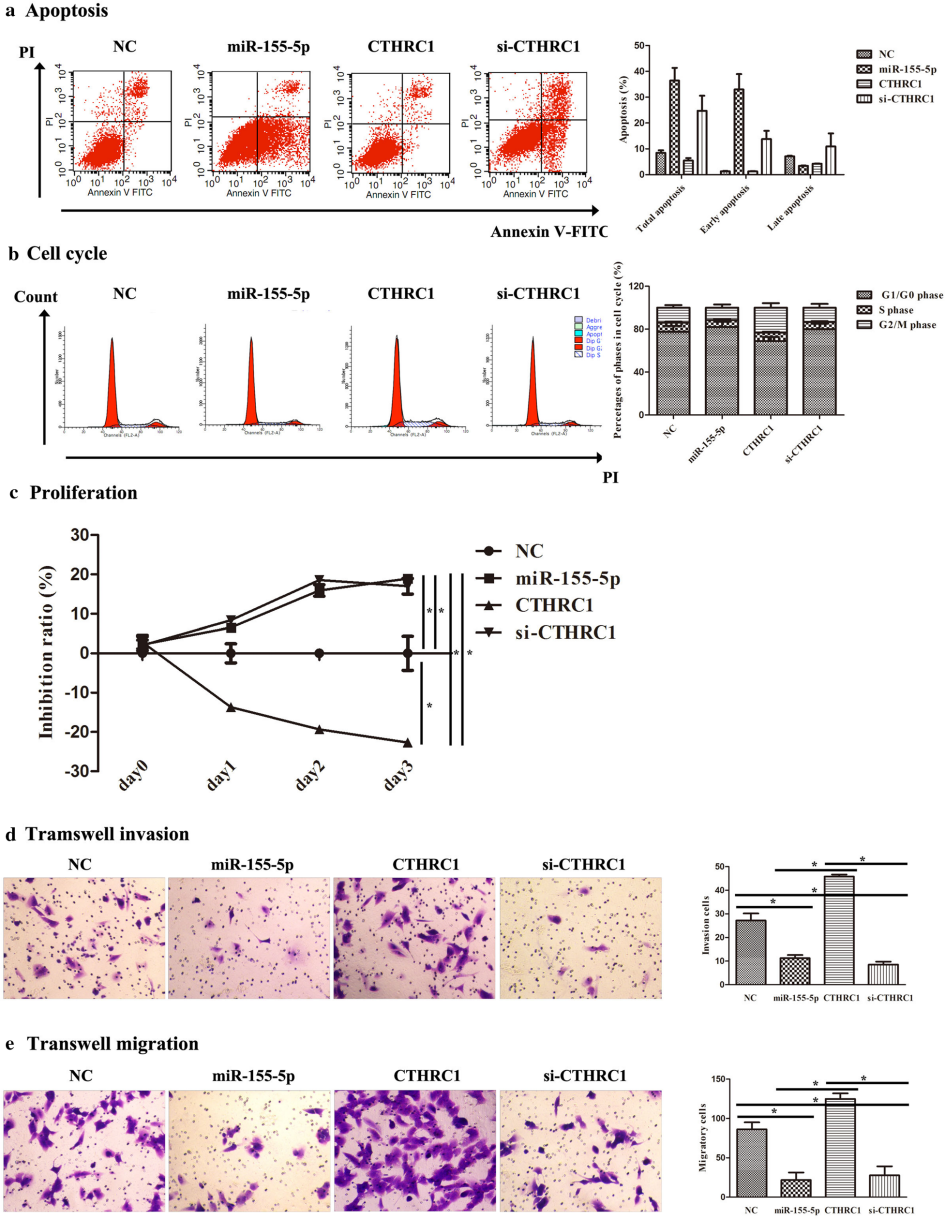
Assessment of the effect of miR-155-5p on apoptosis, cell cycle, proliferation, cell invasion and cell migration by CTHRC1 in HCCLM3 cells. **a** miR-155-5p triggered apoptosis via CTHRC1 in HCCLM3 cells. FACS plots in the left panel show the pattern of apoptosis in representative samples from each group. The statistical histogram is on the right panel. **b** miR-155-5p induced G0/G1 phase cell cycle arrest via CTHRC1 in HCCLM3 cells. Representative cell cycle profiles of each group are presented in the left panel. **c** miR-155-5p promoted cell proliferation via CTHRC1 in HCCLM3 cells. **d** miR-155-5p inhibited cell invasion via CTHRC1 in HCCLM3 cells. Representative images of each group in the Transwell invasion assay. Bar charts show the cell invasion ratio in the right panel. *p < 0.05. Scale bars = 100 μm. **e** miR-155-5p suppressed cell migration via CTHRC1 in HCCLM3 cells. Representative images of each group in the Transwell migration assay. Bar charts show the cell migration ratio in the right panel. *p < 0.05, scale bars = 100 μm

To confirm the effects of miR-155-5p on cell apoptosis and cell cycle progression in HCCLM3 cells, flow cytometry was carried out in our current study. Our data showed that early apoptosis was significantly triggered in cells transfected with the miR-155-5p mimics or the si-CTHRC1 plasmid in comparison with the NC and CTHRC1 groups, while later apoptosis was not remarkably different among the groups (Fig. 4a). Furthermore, cell cycle analysis revealed that the percentage of cells in G1/G0 phase was increased in cells transfected with the miR-155-5p mimics and the si-CTHRC1 plasmid, whereas the percentage of cells in G1/G0 phase was reduced in cells transfected with the CTHRC1-overexpression plasmid (Fig. 4b). Together with the above confirmation in this study that CTHRC1 was a direct target gene of miR-155-5p, these findings indicated that miR-155-5p promoted cell apoptosis and induced G1/G0 phase cell cycle arrest in HCCLM3 cells by inhibiting the expression of its target, CTHRC1.

### miR-155-5p targeting of CTHRC1 suppressed the proliferation, invasion, and migration of HCCLM3 cells

To assess the effect of miR-155-5p and its target CTHRC1 on cell proliferation, we used the CCK8 assay. Compared with the NC group, the overexpression of miR-155-5p or the knockdown of CTHRC1 significantly inhibited cell proliferation; however, up-regulation of CTHRC1 markedly promoted cell proliferation (Fig. 4c). Next, we evaluated whether miR-155-5p and its target, CTHRC1, affect the migratory and invasive behaviors of HCCLM3 cells in vitro (Fig. 4d). The results demonstrated that cells in the miR-155-5p mimic group and the si-CTHRC1 group exhibited obvious decreases in migration and invasion capacities relative to the cells in the NC group. Conversely, augmentation of CTHRC1 dramatically abolished the effects of miR-155-5p and si-CTHRC1 on migration and invasion capacities in HCCLM3 cells. Taken together, we concluded that the suppressive role of miR-155-5p in the proliferation, invasion and migration of HCCLM3 cells might be mediated through targeting CTHRC1.

### miR-155-5p attenuated the growth of tumors through CTHRC1

**Fig. 5:**
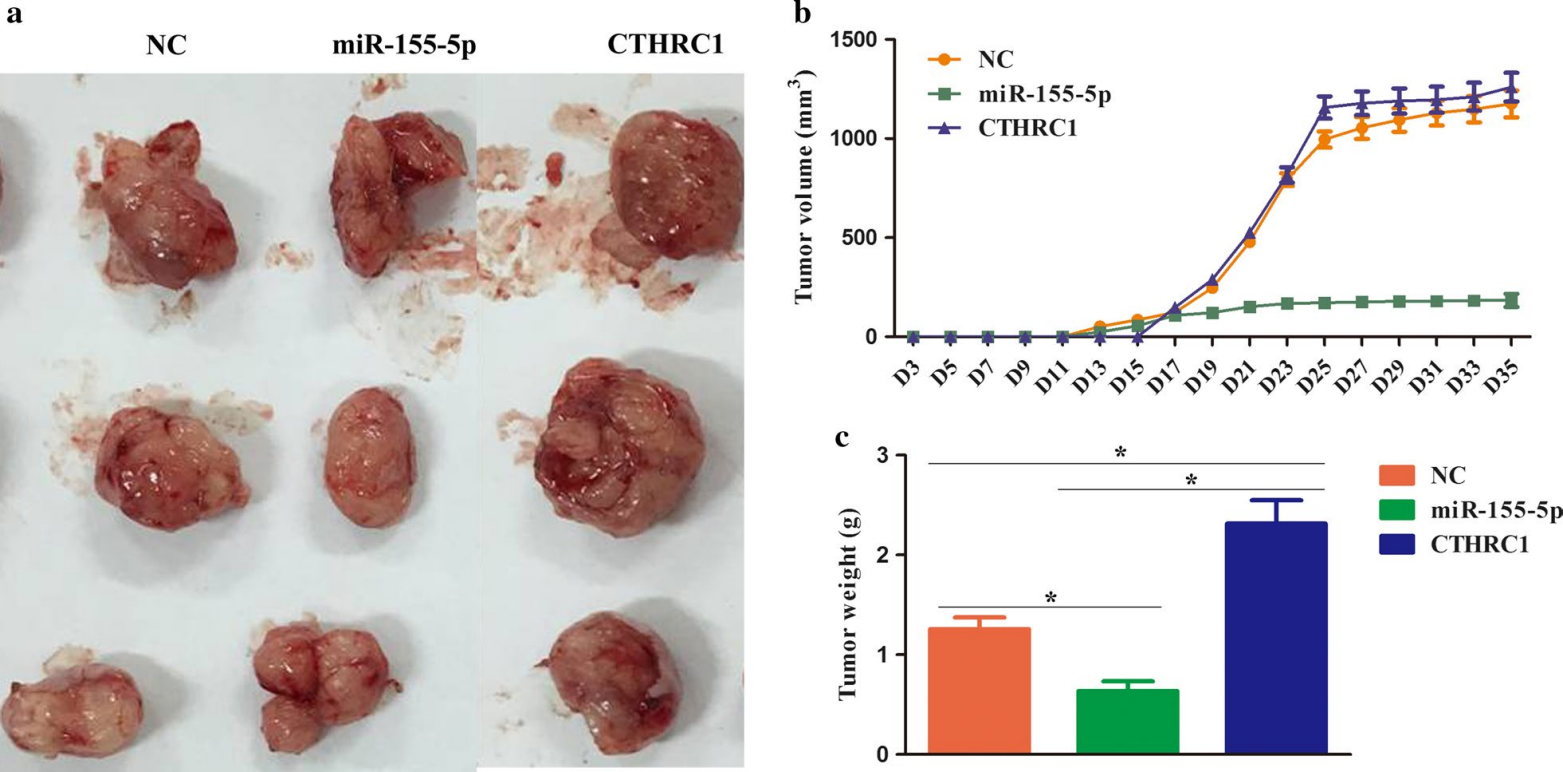
miR-155-5p suppressed tumor growth through CTHRC1 in a nude mouse model. **a** Representative images of tumors in different groups. **b** Growth curve of tumor volume from 3 to 35 days. **c** The tumor weights of each group. ^*^ *p* < 0.05

As seen in Fig. 5, in comparison with the NC group, tumor growth was substantially elevated in the CTHRC1 group and notably suppressed in the miR-155-5p group. Additionally, tumor weight in the CTHRC1 group was greater than that in the NC group, and tumor weight was significantly decreased in the miR-155-5p group when compared to the NC group (p = 0.04, 0.03, 0.02, respectively). Thus, these data suggested that miR-155-5p might delay tumor growth by regulating CTHRC1.

### Effect on the levels of the proteins β-catenin, p-GSK-3β, GSK-3β, Caspase3, Cleaved caspase3, Caspase9, Cleaved PRAP, Bax and Bak by miR-155-5p and its target, CTHRC1

**Fig. 6:**
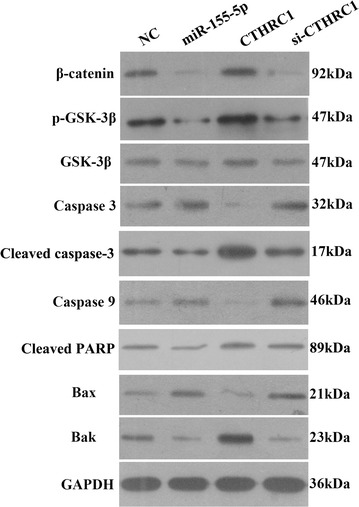
miR-155-5p and CTHRC1 mediated the expression of the proteins β-catenin, p-GSK-3β, GSK-3β, Caspase3, Cleaved caspase3, Caspase9, Cleaved PARP, Bax and Bak as determined by WB assay

The protein expression levels of β-catenin, p-GSK-3β, GSK-3β, Caspase3, Cleaved caspase3, Caspase9, Cleaved PRAP, Bax and Bak in each group were quantitatively analyzed, and the data are presented in Fig. 6. No significant difference in GSK-3β protein expression was found among the four groups. However, the expression levels of β-catenin, p-GSK-3β, and Bak in the miR-155-5p group and the si-CTHRC1 group were remarkably lower than those in the NC group. In addition, the up-regulation of CTHRC1 markedly increased the expression of β-catenin, p-GSK-3β and Bak. Furthermore, Caspase3, cleaved caspase3, Caspase9, cleaved PRAP and Bax protein contents in the miR-155-5p group and the si-CTHRC1 group were notably higher than those observed in the NC group, but these protein expression levels in the CTHRC1 group were obviously reduced compared with those in the other three groups.

## Discussion

Hepatocellular carcinoma remains a serious cancer burden throughout the world [1]. Even though remarkable improvements in the diagnosis and treatment of HCC have been made over recent decades, the prognosis of HCC is still grim, with a dismal 5-year survival rate primarily due to the frequency of metastasis and recurrence, as well as insensitivity to chemotherapy and radiotherapy [2, 19]. This poor prognosis may mainly result from a slow pathological process with gradual changes largely related to genetic and epigenetic alterations [20, 21]. Emerging studies have documented that ample miRNAs could function as tumor suppressors or oncogenes to drive the development and progression of HCC [22]. For example, miR-30a-5p targeted astrocyte elevated gene 1 (AEG-1) to inhibit cell growth and promote the apoptosis of HCC cells [23]; miR-519a accelerated tumor growth via regulation of phosphatase and tensin homolog/phosphatidylinositol-3-OH kinase/protein kinase B (PTEN/PI3K/AKT) signaling in HCC [24]; and miR-125b attenuated epithelial-mesenchymal transition (EMT) by targeting small mothers against decapentaplegic (SMAD)-2 and -4 in HCC [25]. In this study, we initially examined the expression of miR-155-5p in five paired clinical specimens of hepatocellular carcinoma tissues and para-carcinoma tissues and found that miR-155-5p was down-regulated in cancerous tissues compared to juxta-cancerous tissues. However, miR-155, containing two different strands (i.e., miR-155-3p and miR-155-5p), was detected as an oncomir and overexpressed in a series of human malignant cancers, such as colorectal cancer, glioma cancer, oral squamous cancer and even liver cancer [18]. These data were obviously not consistent with our results. The possible reasons for these distinctions include different tumor styles, tissue specificity, various cancer cell lines or ethnic diversity. Additionally, CTHRC1, identified as a novel oncogene in recent years, was also discovered to be up-regulated in several human solid tumors [25], including HCC [26], but the detailed functions of CTHRC1 in HCC have not been fully elucidated. Our data in the present study showed that the expression level of CTHRC1 in HCC tissue was notably higher than that in matched tumor-adjacent tissues, and its protein levels were also confirmed using IHC staining and the WB assay. Moreover, we further predicted the target interaction between miR-155-5p and CTHRC1 and surprisingly found that CTHRC1 might be a potential target of miR-155-5p, which was subsequently verified by the dual-luciferase reporter system, qRT-PCR and WB assays. Additionally, there were opposite expression patterns between miR-155-5p and CTHRC1 in HCC cell lines. Therefore, these findings suggested that ectopic miR-155-5p and CTHRC1 expression might play an important role in the tumorigenesis of HCC.

Next, to investigate the biological roles of miR-155-5p and CTHRC1 in HCC, we first selected one HCC cell line by measuring the gene and protein expression levels of CTHRC1 using qRT-PCR and WB. The results demonstrated that CTHRC1 expression at both the gene and protein levels was highest in HCCLM3 cells; thereby, it was chosen as the cell line for the following experiments. Functionally, elevated miR-155-5p expression induced cell apoptosis and suppressed cell cycle progression and cell proliferation, invasion and migration in HCCLM3 cells. Moreover, the knock-down of CTHRC1 expression had effects similar to the effects of the miR-155-5p mimics on apoptosis, cell cycle progression, proliferation, invasion and migration of HCCLM3 cells. Nevertheless, these effects were mostly reversed in HCCLM3 cells with CTHRC1 overexpression treatment. Cell growth is strictly regulated by apoptosis, the cell cycle and cellular proliferation [27]. Dysregulation of apoptosis, the cell cycle and cellular proliferation originating from miRNA changes is often implicated in the occurrence and development of cancer [28, 29]. For instance, miR-543 inhibited breast cancer cell proliferation, impeded cell cycle progression and enhanced cell apoptosis by directly repressing downstream factors of the mitogen-activated protein kinase/extracellular signal-regulated kinase-2 (MAPK/ERK2) pathway [30]. On the other hand, tumor metastasis, composed of a series of steps including cell invasion, migration and adhesion, was recognized as a common marker in the adverse prognosis of cancers, and these behaviors are intimately linked to the regulation of miRNAs [31]. For example, miR-296 exerted anti-metastatic effects in colorectal cancer by suppressing EMT and the migration and invasion of colorectal cancer cells [32]. Thus, we concluded that miR-155-5p negatively mediated tumor growth and metastasis of HCC in vitro by CTHRC1 modulation. Furthermore, a great deal of literature has demonstrated that the elevated expression of CTHRC1 was ubiquitously associated with cancer proliferation, invasion, migration, adhesiveness and metastasis and ultimately contributed to the initiation, promotion and progression of cancers, including pancreatic cancer, epithelial ovarian cancer, non-small cell lung cancer and colorectal cancer [33], which further validated our above stated conclusion. Finally, a xenograft model of BALB/c nude mice was established to certify the impacts of miR-155-5p and CTHRC1 on tumor formation in vivo. It was observed that the tumor volume and weight in the miR-155-5p mimic group were markedly increased in comparison with those in the CTHRC1 group, indicating that miR-155-5p functioned in a tumor-suppressive role in HCC by inhibiting CTHRC1 expression and that CTHRC1 functioned in a tumor-promoting role in HCC by miR-155-5p regulation.

To further excavate the underlying molecular mechanism of miR-155-5p and CTHRC1 in HCC growth and metastasis, we eventually identified their downstream signaling pathway. Previous studies revealed that the forced expression of CTHRC1 facilitated cancer cell proliferation, invasion and metastasis in epithelial ovarian cancer [34] and non-small cell lung cancer [35] in vitro through the Wnt/β-catenin signaling pathway. Moreover, it is well known that activation of the Wnt/β-catenin pathway plays a fundamental role during the early progression of metastasis and tumor growth [35–37]. During the tumorigenesis process, the triggering of Wnt could prevent GSK-3β from activation and maintain β-catenin stabilization, which are highly correlated with tumor EMT [38, 39]. Thus, exploring the GSK-3β-involved Wnt/β-catenin pathway could help us better understand the potential mechanism of miR-155-5p and CTHRC1 in HCC growth and metastasis. Gain- and loss-of-function CTHRC1 experiments demonstrated that metastasis-related proteins (i.e., β-catenin, p-GSK-3β, and GSK-3β) and the anti-apoptosis-related protein Bak were significantly increased and that pro-apoptosis-related proteins (i.e., Caspase3, Caspase9 and Bax) were remarkably decreased in the CTHRC1 group, while the si-CTHRC1 group presented an opposite expression pattern for these proteins, which was in accord with the miR-155-5p group in HCCLM3 cells. Furthermore, cleaved caspase3 and cleaved PRAP, which are considered markers of apoptosis, were also up-regulated in the miR-155-5p and si-CTHRC1 groups but down-regulated in the CTHRC1 group compared to the NC group. Collectively, we speculated that HCCLM3 cells secreted CTHRC1 into the tumor microenvironment by down-regulating miR-155-5p and then attenuated cancer cell growth and metastasis by activating GSK-3β-involved Wnt/β-catenin signaling.

## Conclusion

We demonstrated for the first time that miR-155-5p and CTHRC1 presented an inverse expression pattern in HCC patients and that their functional roles in the development and progression of HCC were exerted by regulating GSK-3β-involved Wnt/β-catenin signaling. Hence, these data implied that miR-155-5p and CTHRC1 were considered not only new molecular biomarkers in predicting the aggressive biology of HCC but also novel therapeutic targets to guide effective treatment for HCC patients.

## Additional file

**Additional file 1: Figure S1.** The binding site between miR-155-5p and CTHRC1 was predicted by bioinformatics analysis using TargetScan.

## Abbreviations

HCC: hepatocellular carcinoma (HCC)
BSA: bovine serum albumin
CTHRC1: collagen triple helix repeat containing 1
IHC: immunohistochemistry
PDCD4: programmed cell death 4
RIPA: radioimmunoprecipitation assay
qRT-PCR: transcription-polymerase chain reaction
CCK8: Cell Counting Kit-8
PVDF: polyvinylidene difluoride
