# Supplementary figures and images for: miR-155-5p modulates malignant behaviors of hepatocellular carcinoma by directly targeting CTHRC1 and indirectly regulating GSK-3β-involved Wnt/β-catenin signaling
Source: Cancer Cell Int. 2017 Dec 8;17:118. doi: 10.1186/s12935-017-0469-8 (PMC5721693; doi:10.1186/s12935-017-0469-8)

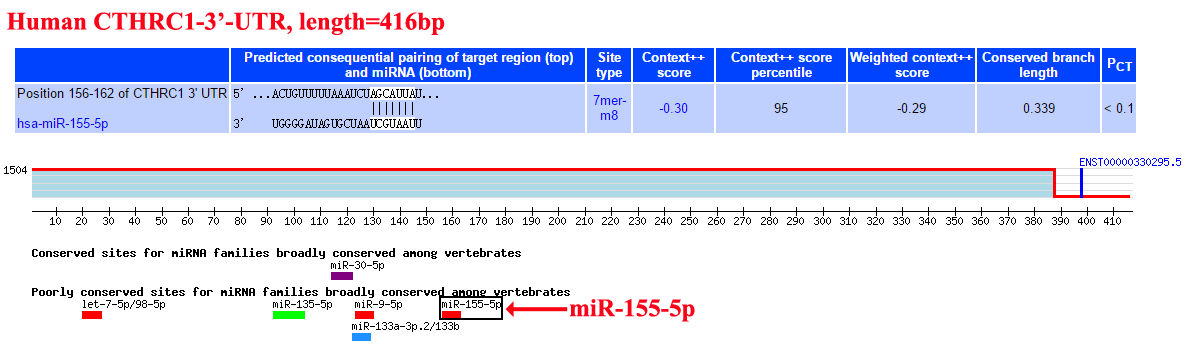

Supplement: Supplementary file 1 — Additional file 1: Figure S1. The binding site between miR-155-5p and CTHRC1 was predicted by bioinformatics analysis using TargetScan. [file 12935_2017_469_MOESM1_ESM.tif]
